# Preclinical characterisation of changes in cardiac function and circulating biomarkers following differential irradiation of thoracic volumes

**DOI:** 10.3389/fonc.2025.1623753

**Published:** 2025-06-27

**Authors:** Refik Kuburas, Mihaela Ghita-Pettigrew, Gerard M. Walls, Brianna N. Kerr, Kathryn H. Brown, Cecilia Facchi, Alan McWilliam, Marcel van Herk, Kaye J. Williams, Karl T. Butterworth

**Affiliations:** ^1^ Patrick G. Johnston Centre for Cancer Research, Queens University Belfast, Belfast, United Kingdom; ^2^ Division of Cancer, University of Manchester, Manchester, United Kingdom; ^3^ Department of Radiation Related Research, University of Manchester, Manchester, United Kingdom; ^4^ Division of Pharmacy and Optometry, University of Manchester, Manchester, United Kingdom

**Keywords:** radiotherapy, preclinical model, cardiotoxicity, heart irradiation, lung irradiation, cardio-pulmonary, radiation-induced cardiac fibrosis, circulating biomarkers

## Abstract

**Introduction:**

The heart and lungs are critical organs at risk in patients receiving radiotherapy for thoracic tumours. Preclinical studies in rat models have provided evidence indicating consequential effects of lung radiation on the heart through vascular remodelling which leads to pulmonary arterial hypertension. In this study, we aimed to assess the impact of lung irradiation on a long-term model of cardiac base irradiation that recapitulates clinical observations of the heart base as a radiosensitive region and to understand relationships between cardiopulmonary irradiation and circulating cytokines profiles.

**Methods:**

Female C57BL6J mice were irradiated under CT image-guidance targeting the heart base, right lung or co-irradiation of the heart base and the right lung. Mice were monitored by transthoracic echocardiography for 50-weeks after irradiation with lung histology and cytokine profiling at 10 and 50 weeks.

**Results:**

Lung and heart co-irradiation leads to small changes in the cardiac function and histological changes in the right lung with distinct changes in serum cytokines for different irradiated volumes compared to heart irradiation.

**Discussion:**

In contrast to previous studies in rat models, these data demonstrate a minimal contribution of lung irradiation to cardiac response in this model. Understanding the potential interplay between the heart and lungs is important in the context of optimising cardiac dose distributions that may increase lung doses and minimising the impact of lung dose on cardiac function.

## 1 Introduction

Radiation-induced normal tissue toxicities are a complex challenge in the management of patients with non–small cell lung cancer (NSCLC) treated with radiotherapy (RT) (1). During irradiation of the thorax, the proximity of the heart and lungs results in coincidental dose to both tissues. Whilst cardiac and pulmonary toxicities are independently well established, relatively little is known concerning potential interactions between these organs that could impact cumulative toxicities.

Treatment-related toxicities in the lung in the form of acute radiation pneumonitis (RP) or late occurring fibrosis (RF) are important dose limiting factors that directly impact patient outcomes and quality-of-life after treatment (2). In the heart, radiation-induced cardiotoxicity (RICT) can present as a range of pathologies including ischemia, arrhythmia, heart failure, valvulopathy, or pericardial disease months or years post-treatment (1, 3, 4). Historically, treatment planning has aimed to minimise the mean dose to both the heart and lung volumes based on the assumptions that each tissue is uniformly radiosensitive and responses are spatially independent (5). However, evidence suggests that distinct spatial dose volume effects in the lungs (6, 7) and heart (8, 9) can impact the probability of complications in these organs. Clinical studies suggest a strong spatial dependency with dose to specific cardiac substructures (10) associated with poor survival and cardiac outcomes (10, 11) yet the optimum dose constraints for cardiac substructures remain to be determined (10).

Understanding the complexity of spatial dose volume relationships in normal tissues highlights potential opportunities for optimisation by avoiding radiosensitive subvolumes to reduce the probability of complications without compromising target coverage or exceeding dose constraints in other organs at risk (OARs). This approach is being explored in lung cancer by defining a cardiac avoidance area (CAA) based on evidence linking excess dose to subvolumes and overall survival (12). An important consideration is the impact of avoidance on other OARs yet treatment planning evidence suggests that such an approach can significantly reduce heart dose with no significant increases in doses to other thoracic OARs including the lungs (13).

Previous experimental studies in rat models have provided evidence showing potential interactions between the heart and lungs following radiation exposure (14–16). Irradiation of the heart results in changes in the cardiac vasculature and myocardium leading to cardiac dysfunction which also causes pulmonary interstitial oedema. Lung irradiation can indirectly impair cardiac function through radiation-induced pulmonary hypertension and pulmonary perivascular oedema. In addition, co-irradiation can enhance cardiac dysfunction via both mechanisms (14, 17). Further studies are needed to characterise the interplay between the cardiac and pulmonary systems, and to better understand the spatial dose volume relationships in these organs leveraging the advantages of image-guided irradiators in preclinical models (18–21).

We have established and characterised a translationally relevant mouse model of heart base irradiation that recapitulates clinical observations identifying the heart base as a radiosensitive subvolume associated with functional loss (22–24). In the current study, we aimed to apply this model to investigate potential interactions between the heart and lungs following radiation exposure by assessing changes in cardiac function, lung histology and circulating biomarkers following irradiation of the heart or co-irradiation of the heart and lungs.

## 2 Materials and methods

### 2.1 Animals and maintenance

Female C57BL6J mice, 12–16 weeks-old (Charles River Laboratories, Oxford, UK) were housed under controlled conditions (12hr light-dark cycle, 21°C) in standard caging with 3–5 littermates and received a standard laboratory diet (Teklad, Envigo, UK) with water a*d libitum*. Mice were randomly assigned to experimental groups prior to irradiation. Animal numbers were chosen to give power to detect differences in myocardial performance index (MPI) of 25% with a power of 80% for a 2-sided equality test with a significance threshold of 0.05 which required at least six mice per group. In this study we used 8 mice per group for each timepoint, with aged matched controls at week 50.

Mouse weight was monitored throughout the experiment, with no deviation outside the tolerated weight loss of <15%. All animal research and experiments carried out at Queen’s University Belfast were approved by the local Animal Welfare Ethical Review Board (AWERB) and a Department of Health Project License (PPL 2935). All experimental procedures were carried out in accordance with the Home Office Guidance on the Operation of the Animals (Scientific Procedures) Act 1986, published by His Majesty’s Stationary Office, London. Animal studies are reported in compliance with the ARRIVE guidelines and suggested reporting requirements for preclinical cardiac irradiation studies (25, 26).

### 2.2 Irradiation protocol

**Figure 1:**
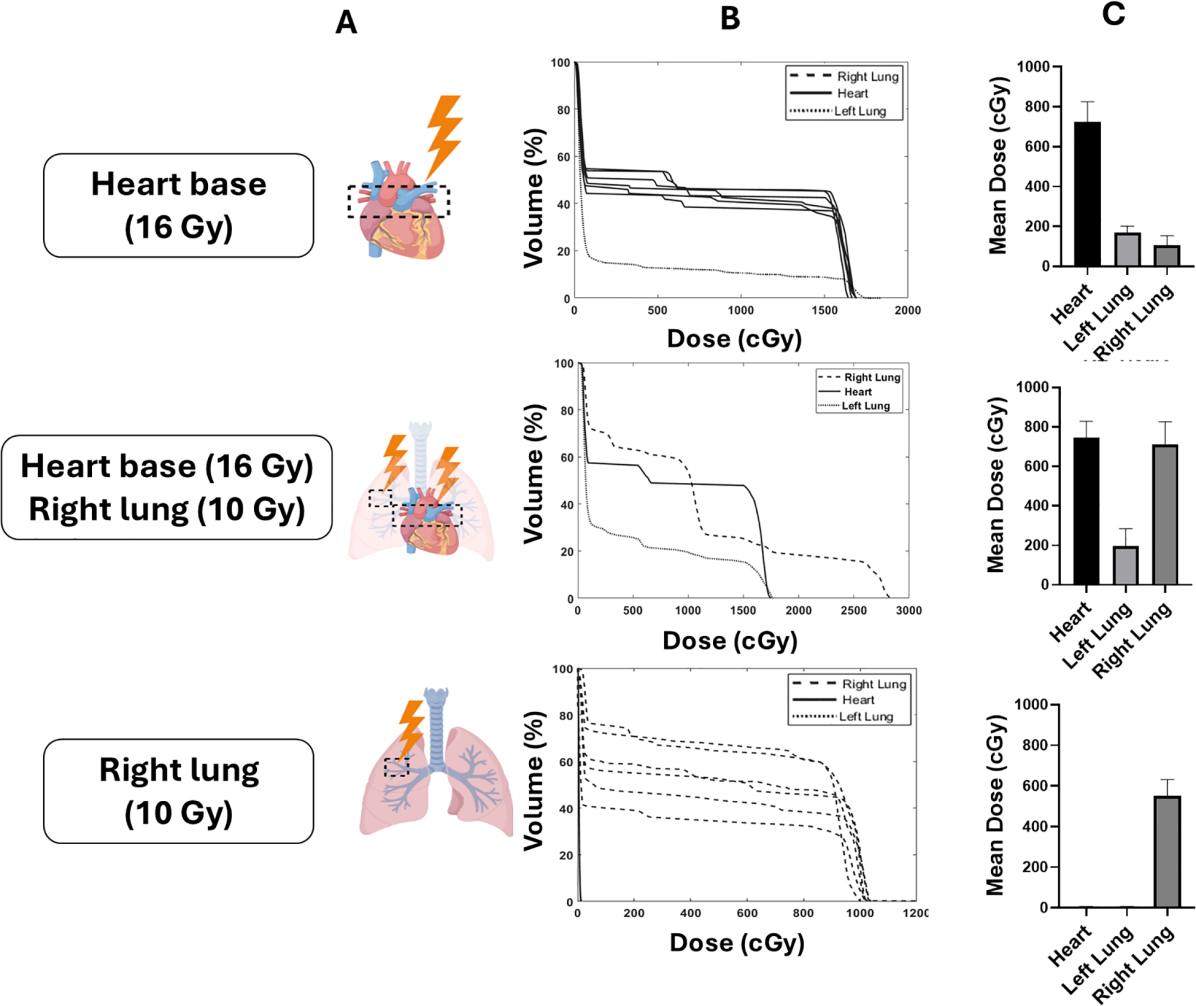
Schematic overview of the experimental configurations targeting different thoracic volume in the heart, right lung and co-irradiation. Mice were randomized to heart base irradiation (16 Gy), right lung irradiation (10 Gy), or co-irradiation of the heart base (16 Gy) and right lung (10 Gy) **(A)**. Dose volume histograms (DVHs) and mean heart doses (MHD) for each of the targets are shown in **(B, C)**, respectively.

Prior to irradiation mice were anaesthetised with ketamine (100 mg/kg) and xylazine (10 mg/kg) by intraperitoneal injection. Mice were randomized prior to irradiation to either a control non-irradiated group, heart base irradiation with 16 Gy, right lung irradiation with 10 Gy or a combined heart base (16 Gy) and right lung (10 Gy) irradiation. Mice were irradiated with 220 kVp X-rays under cone beam computed tomography (CBCT) image guidance using a SARRP (Xstrahl Life Sciences) calibrated using the Institute of Physics and Engineering in Medicine and Biology (IPEMB) code of practice (27). For heart base irradiation, the cranial third of the heart was localised using CBCT imaging and an anterior-posterior field arrangement was created using a 3 x 9 mm collimator with a dose rate of 2.67 ± 0.11 Gy/min as previously described (22). Dose to the upper region of the right lung was delivered using a 5 x 5 mm collimator at a dose rate of 2.75 ± 0.23 Gy/min in a similar anterior-posterior beam geometry. Dose-volume histograms (DVHs) were retrospectively calculated for each animal (Muriplan, Xstrahl Inc, Suwannee, GA, USA). A schematic overview of the study including the DVHs and endpoints is shown in **Figure 1**.

### 2.3 Transthoracic echocardiography

TTE was performed at baseline and 50-weeks after irradiation using Vevo770^®^ ultrasound system, with a 45 MHz RMV707B scan head (FUJIFILM, VisualSonics Inc. ON, Canada). Mice were anaesthetised using inhalant isoflurane (3% for induction, and 1.5% during imaging). Left ventricular (LV) systolic and diastolic metrics were measured in triplicate for each mouse. LV ejection fraction (EF) and fractional shortening (FS) were calculated from the M-mode parasternal short-axis view, as previously described (28). Pulse-wave Doppler imaging was used to measure the early and late filling ratio (E/A ratio). MPI was calculated as previously described (28). Observers were blinded to experimental groups during data collection and analysis.

### 2.4 Histopathological analysis of the irradiated right lung

Lung were harvested at 10 and 50-weeks after irradiation. After fixation, samples were processed, paraffin embedded and cut into 5 µm sections for histological staining and evaluation. Pulmonary perivascular and interstitial oedema were assessed using the semi-quantitative scoring previously reported (17). Perivascular oedema was scored between 0-2 (2 indicating severe perivascular oedema) for at least 5 large vessels and 20 small vessels across the right lung. For interstitial oedema, scores from 0-3 (3 severe interstitial oedema) were given according to the level damage severity across 4 different fields of view (400 x 400 µm) across the right lung (17). Pulmonary vessel wall thickness was measured for 30 randomly chosen vessels across in the upper right lung as previously described (14). Fibrosis was measured by type I collagen staining using Masson’s Trichrome (Abcam, ab150686) as outlined in **Supplementary Material S1**. Scores were recorded from 6 different regions within or outside of the irradiated field of the heart or right lung for 4 mice per experimental group and ≥2 age matched control animals. Lung fibrosis was quantified using Matlab R2022a as previously described (29).

### 2.5 Analysis of cytokine expression and pathway enrichment

Mouse XL Proteome Profiler Cytokine arrays (ARY028, R&D Systems, UK) were performed to simultaneously characterise the expression of 111 cytokine targets. Each cytokine was present in duplicate on the membrane. Serum used for analysis was isolated from blood samples at weeks 10 and 50 from irradiated mice alongside age-matched controls. Cytokine arrays were performed according to the manufacturer’s instructions using pooled serum samples for each treatment group (control n=2, heart n=6, lung n=6, heart and lung n=6). Membranes were developed using the GBox Imager and GeneSys software (V1.4.60) (Synotics Ltd, Cambridge, UK). Quantification of signal intensities was performed with GeneTools software (V4.3.14) (Synotics Ltd, Cambridge, UK), and densitometry values for spots were normalised to that of the age-matched controls. Cytokine arrays were completed in one independent experiment. Proteins which satisfied a threshold of *>* 2- and *<* 0.5-fold change were considered differentially expressed. These proteins were transformed into their corresponding gene names for pathway enrichment analysis using Enrichr (30).

### 2.6 Statistical analysis

Data are presented as the average for the entire experimental arm ± standard error. Error bars represent the standard error of the mean (SEM). Statistical analysis was performed using student’s t-test unless otherwise stated. Statistical significances are represented as *p* < 0.05 = *; *p <*0.01 = **; *p <*0.001 = ***; and *p <*0.0001 = ****. All bar graphs, heatmaps, and principal component analysis (PCA) plots were created using GraphPad Prism (V9.2.0).

## 3 Result

### 3.1 Cardiac function is impaired by heart base irradiation and is not impacted by lung co-irradiation

**Figure 2:**
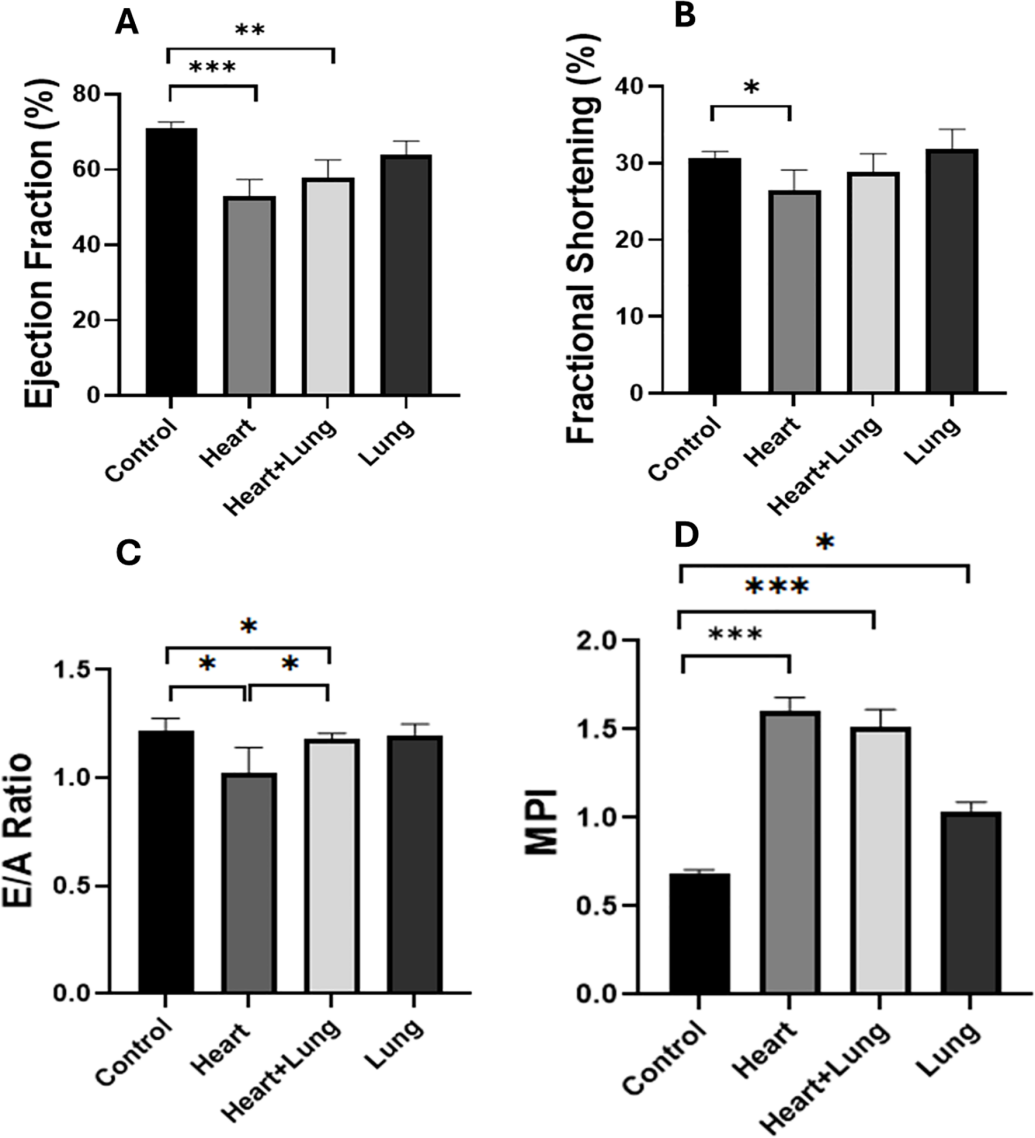
Longitudinal variations in cardiac functional parameters following irradiation of different thoracic volumes at 50 weeks after irradiation (Ejection fraction, EF, **(A)** Fractional shortening, FS. **(B)** E/A Ratio, **(C)** Myocardial Performance Index, **(D)**]. No significant changes in parameters were observed at 10 weeks after irradiation (**Supplementary Figure S1**). Data presented are an average of 6 mice per treatment group ± SEM against age-matched control values. Significance values were classified as *p < 0.05, **p < 0.01 and ***p < 0.001.

Cardiac systolic and diastolic parameters were assessed following the irradiation of different thoracic volumes. No statistically significant changes in cardiac function parameters were observed between control and irradiated mice at 10 weeks after irradiation (**Supplementary Figure S1**). At 50 weeks after irradiation, several changes in cardiac function were observed between control and irradiated animals (**Figure 2**). Systolic function measured by EF was significantly lower after irradiation of the heart base and co-irradiation of the heart base and right lung (p<0.004) compared to control animals. A small but significant decrease in FS was observed only for heart base irradiation compared to control animals (p=0.036). Diastolic function measured by the E/A ratio was significantly lower after heart base and co-irradiation of the heart base and right lung (*p* =0.038). MPI is a measure of the overall performance of the heart integrating both systolic and diastolic function that increases with cardiac dysfunction. MPI was significantly increased in all irradiated groups with much higher increases in the heart base and co-irradiation groups compared to the control group (p<0.001). Overall, these data show a significant effect of heart base irradiation on cardiac function with co-irradiation of the right lung having minimal impact on the observed responses at 50 weeks after irradiation.

### 3.2 Heart and lung co-irradiation modulates histological changes in the lung

**Figure 3:**
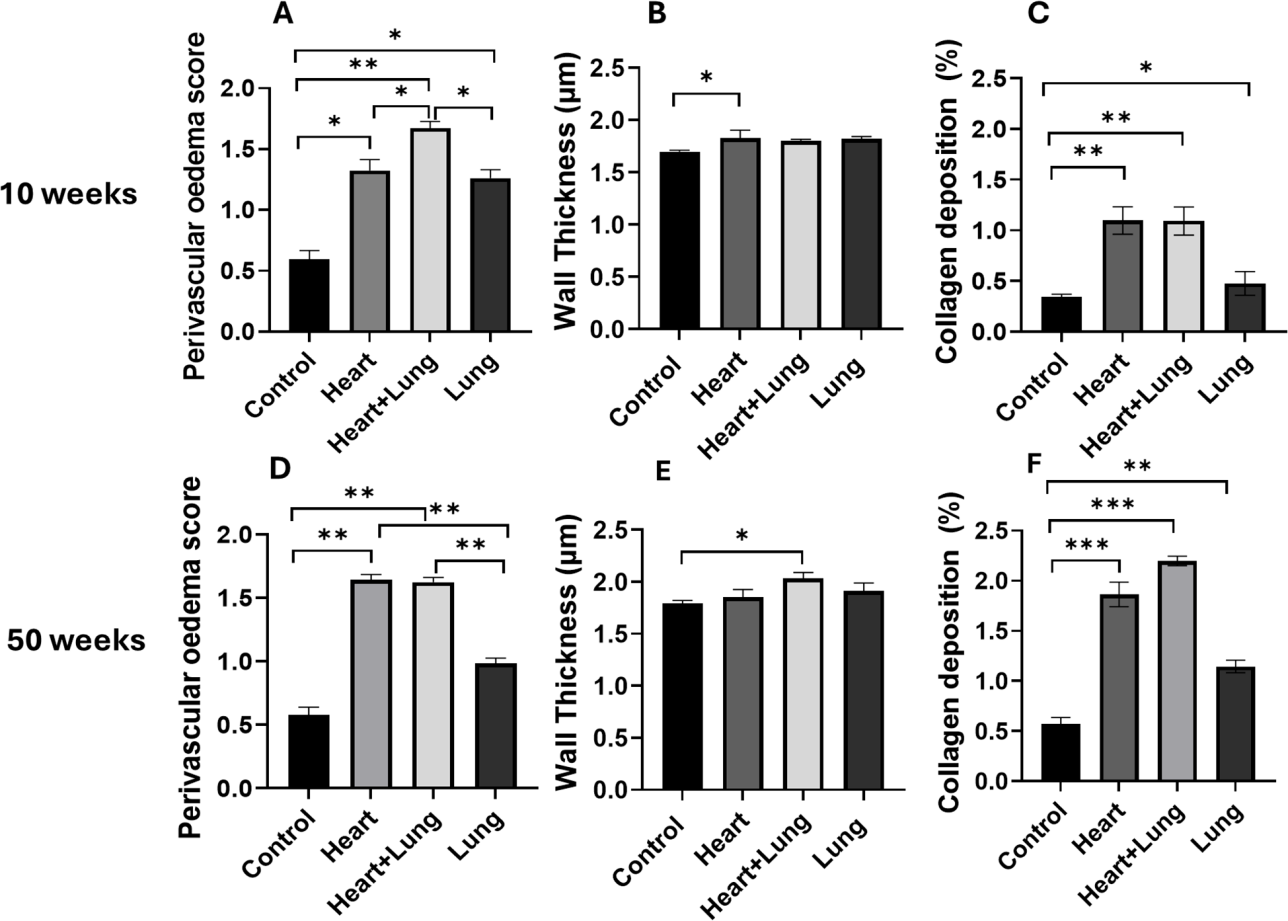
Histological changes and collagen levels in the irradiated right lung at 10 and 50 weeks following irradiation of different thoracic volumes. The levels of perivascular oedema and pulmonary vessel wall thickness were assessed following H & E staining **(A, B, D, E)**. Fibrosis was assessed by type I collagen staining **(C, F)**. Data presented are an average of 6 mice per treatment group (n = 2 for 50-week control group) ± SEM against age-matched control values. Significance values were classified as *p < 0.05, **p < 0.01 and ***p < 0.001. Scale bar = 50 µm.

Histological changes were assessed in the irradiated right lung at 10 and 50 weeks after the irradiation of different thoracic target volumes (**Figure 3A-F**). At 10 weeks after irradiation, all groups showed a significant increase in pulmonary oedema compared to control animals (p<0.05) with the largest increase observed in the co-irradiation group. Irradiation of the heart and lungs as individual targets showed comparable levels of response that were not significantly different (p > 0.05, Panel A). A small but significant increase in pulmonary vessel wall thickness was only detected following heart base irradiation. Heart and heart lung co-irradiation showed small but significant increases in fibrosis that was higher than irradiation of the lung as a single target (Panel C). Similar trends were observed at 50 weeks after irradiation showing significant increases in pulmonary oedema compared to control animals (p<0.01) with similar increases in the heart and co-irradiation groups. A small but significant increase in pulmonary vessel wall thickness was only detected following co-irradiation whilst the levels of fibrosis were significantly higher in the heart and co-irradiation groups. These data suggest small but significant histological changes in lung that are dependent on the irradiation of the heart. Represantative images from histology analysis are shown in **Supplementary Figure 2**.

### 3.3 Heart and lung co-irradiation leads to differential changes in the levels of circulating cytokines

**Figure 4:**
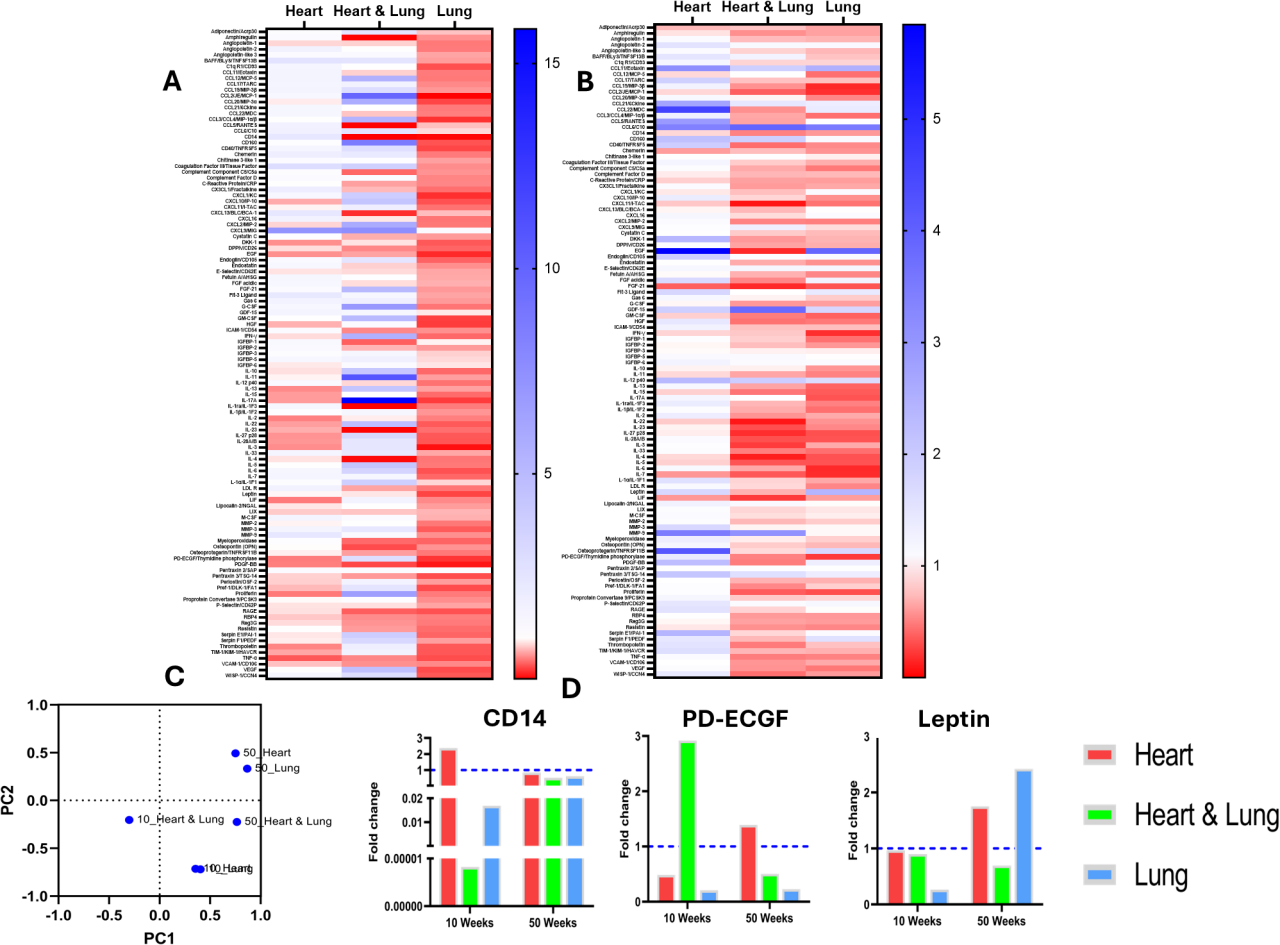
Expression of circulating inflammatory cytokines at 10 and 50 weeks following irradiation of different thoracic volumes. Heatmaps of fold changes in cytokine expression compared to age-matched controls are shown at 10 **(A)** and 50 **(B)** weeks after irradiation of the heart, heart and lung co-irradiation and the right lung. A principal component analysis (PCA) plot of the samples is shown in **(C)** Changes in the levels of biomarkers with known cardio-pulmonary functions are shown in **(D)**.

Cytokine analysis was performed on serum samples obtained at 10 and 50-weeks after irradiation (**Figure 4**). Heatmaps show the fold changes in the expression of 111 cytokines compared to age-matched controls at the respective timepoints for each target volume (**Figures 4A, B**). These data highlight distinct changes for each of the irradiated volumes and at each time point with an overall trend showing decreased cytokine expression following irradiation of the lungs compared to the other volumes. PCA was used to identify similarities in modulated cytokine expression between the different irradiated volumes (**Figure 4C**). The PCA plot shows clustering of the individual heart and lung volumes within each timepoint suggesting similar cytokine modulatory effects. The observed changes occurred predominantly across PC2. In contrast, combined heart and lung irradiation has a unique effect on cytokine expression at both timepoints, reflected by the lack of clustering and the individuality of these data points. These changes occurred across PC1.

Changes in specific cytokine levels demonstrated differential responses following irradiation of the different target volumes (**Figure 4D**). In particular, a clear difference was observed in the levels of CD14 for the different irradiated volumes. At 10 weeks, CD14 showed a >2-fold upregulation following irradiation of the heart yet the level was several orders of magnitude lower following co-irradiation or irradiation of the lung only. However, at 50 weeks, no major changes in CD14 were observed. Platelet-derived endothelial cell growth factor (PD-ECGF) also showed a pronounced volume dependent response. At 10 weeks, PD-ECGF showed > 2-fold upregulation following co-irradiation yet the levels were down regulated following irradiation of the heart or lung. At 50 weeks, the levels of PD-ECGF were lower in the co-irradiation group, elevated in the heart group and unchanged in the lung group compared to the level at 10 weeks. At 10 weeks, the levels of leptin remained unchanged compared to controls in the heart and co-irradiation groups but were lower in the lung irradiated group. At 50 weeks, the levels increase close to 2-fold following heart and lung irradiation as single targets but decreased in the co-irradiation group. Overall, these data highlight distinct changes in the circulating inflammatory proteome that are dependent on the irradiated target volume and time after irradiation.

**Figure 5:**
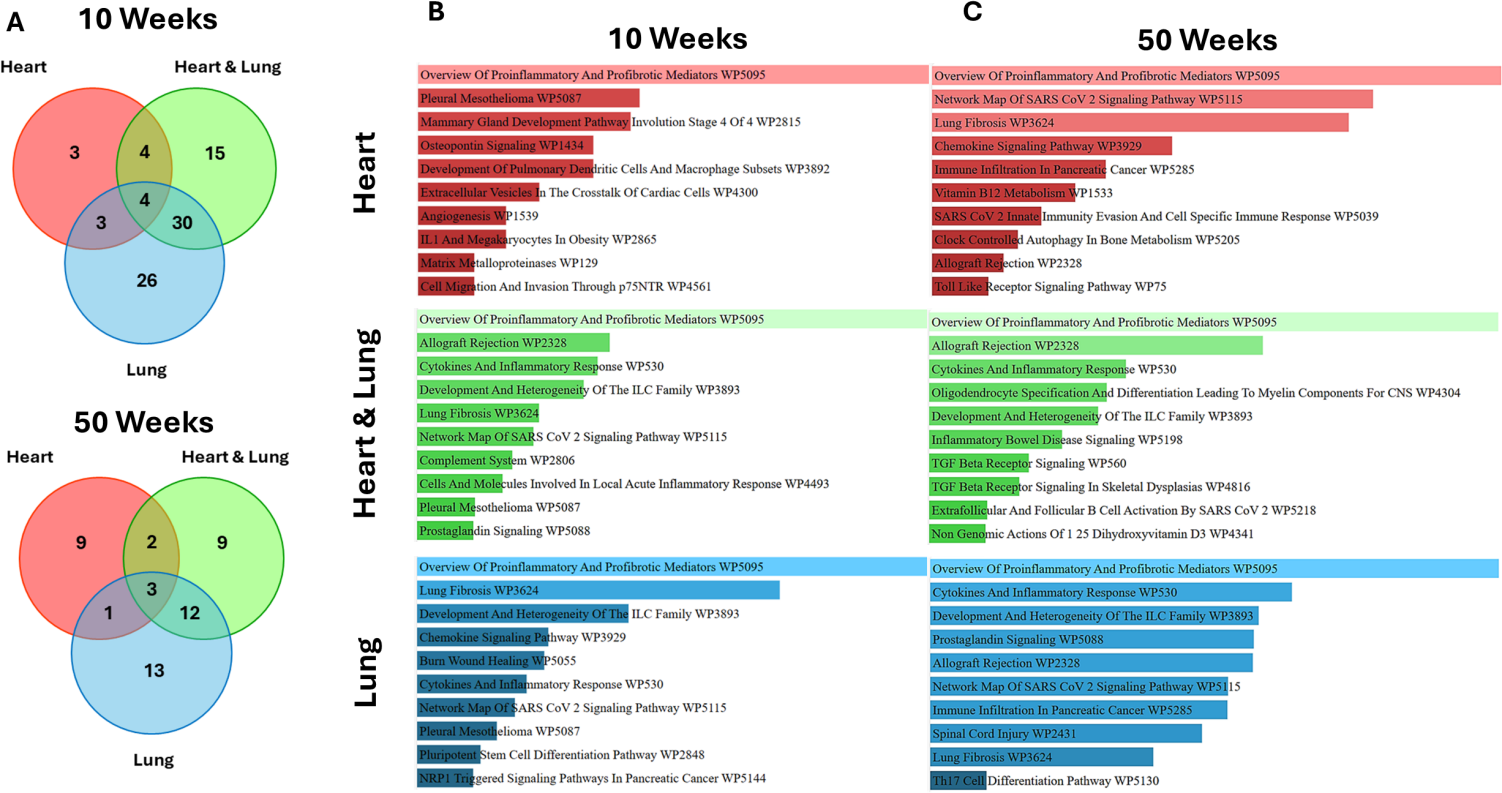
Analysis of volume-specific cytokines and associated biological pathways at 10 and 50 weeks following irradiation of different thoracic volumes. Cytokines showing > 2-fold changes for the different volumes at 10 and 50 weeks **(A)** with pathway enrichment analysis at the respective timepoints **(B, C)**.

Differential cytokine analysis was performed by applying a threshold of > 2-fold changes at 10 and 50 weeks (**Figure 5A**). At 10 weeks, 3, 15 and 26 cytokines were found to be volume-specific for the heart, heart and lung, and lung irradiations, respectively. At 50 weeks, 9, 9 and 13, cytokines were found to be volume-specific for the heart, heart and lung, and lung, respectively. The identified volume specific cytokines were then used for pathway enrichment analysis (**Figures 5B, C**). For all of the irradiated volumes at 10 and 50 weeks, the most enriched pathway was proinflammatory and profibrotic mediators. At 10 weeks, lung fibrosis was enriched following lung only and co-irradiation but not for heart irradiation, however, at 50 weeks, lung fibrosis was enriched in the heart and lung irradiation groups. These data support the underlying biological pathways involved in the inflammatory response of the respective tissues and suggest a potential interaction between the heart and lung that acts to suppress fibrosis when the organs are co-irradiated.

## 4 Discussion

The heart and lungs are critical organs at risk in patients receiving RT for thoracic cancers. Current treatment planning practice considers the risk of complications in these tissues independently according to organ specific dose constraints. Previous preclinical studies in rat models have demonstrated potential interactions between the heart and lungs following lung irradiation characteristic of pulmonary arterial hypertension (PAH). This process is driven by pulmonary vascular remodelling leading to arterial hypertension and increased burden on the right ventricle (14, 17). In the current study, we aimed to assess the impact of lung irradiation on cardiac radiation response and to identify changes in the circulating biomarkers following irradiation of the heart or co-irradiation of the heart and lungs.

Using CT image guidance to target the base of the heart, our data showed distinct functional loss of systolic and diastolic parameters and MPI changes at 50 weeks after irradiation consistent with our previous observations in this model (22). These changes may be due to the targeting of critical structures located in the base of the heart including the coronary arteries, pulmonary arterial trunk and pulmonary veins, or pacemaker tissues including the sinoatrial and atrioventricular nodes. Compared to age matched control animals, co-irradiation of the heart base and right lung resulted in small but significant changes in EF, E/A ratio and MPI that were smaller than those observed for irradiation of the heart alone. The unexpected small increases in EF, FS and E/A ratio compared to heart irradiated animals suggests a potential sparing of cardiac function when the right lung is irradiated. These observations were supported by an upwards trend in MPI indicating improved global cardiac performance for co-irradiation compared irradiation of the heart. These data suggest subtle alterations in the timing and overall efficiency of the cardiac cycle due to subclinical dysfunction, combined systolic and diastolic impairment, or regional changes that are not captured by global measures. In contrast to previous studies in rats (14, 15, 31), these findings show a minimal contribution of lung irradiation to cardiac response in this model. Further validation is required potentially using cardiac strain to measure deformation of the myocardium during the cardiac cycle. We have recently demonstrated this approach as an early predictive biomarker of cardiac function (23), however, this technology was not available in our laboratory at the time of investigation.

Histological changes in the lung showed small but variable changes in perivascular oedema, wall thickness and collagen levels following irradiation of the different volumes at 10 and 50 wks. Minimal changes in vessel wall thickness at were observed at both times points. Whilst some of the observed changes are statistically significant, they are < 2% in most cases. These data suggest minimal impacts of heart irradiation on the histology of the lung and are likely to have limited functional consequences on heart through the development of PAH in this model.

The mechanisms underlying the indirect effects of lung irradiation on the heart remain to be fully understood. Given the central role of inflammatory cytokines in mediating radiation-induced pneumopathy (32, 33), cytokine profiling was conducted on serum samples at 10 and 50 weeks after irradiation to characterise changes in circulating proteins and to identify potential mechanisms of response. PCA showed clustering of samples for the individual heart and lung volumes at each timepoint suggesting similar cytokine modulatory effects. In contrast, combined heart and lung irradiation showed a distinct expression profile at both timepoints highlighting volume specific responses presented as Venn diagrams in **Figure 4**.

At 10 weeks, differential expression analysis identified cytokine changes specific to each volume and identified upregulation of CD14 following heart radiation. CD14 is a glycoprotein receptor that has been shown to promote macrophage activation and is closely linked to myocardial dysfunction and adverse remodelling (34, 35). In the plasma, soluble CD14 (sCD14) arises from proteolytic cleavage of the membrane-bound form (36). Clinical studies have reported significant associations between sCD14 levels, atherosclerotic cardiovascular disease and heart failure (37). The association of sCD14 with cardiac radiation response has not been reported and our data suggest sCD14 as a potential biomarker of RICT. Following lung irradiation, leptin was identified as differentially down-regulated. Leptin is a hormone secreted by adipocytes that has been shown to promote lung cancer progression and therapeutic resistance and thus may be considered a therapeutic target following lung-targeted RT (38–40). Finally, co-irradiation of the heart and lung resulted in differential upregulation of PD-ECGF. PD-ECGF is a pro-angiogenic factor that is involved in constrictive vascular remodelling in the heart (41). This suggests that PD-ECGF may be an important molecule involved in radiation response of the heart and lung. Whilst these exploratory data are informative in characterising cytokine driven changes following thoracic irradiation, further validation is required in clinical samples.

To further understand the biological pathways involved follow irradiation, pathway enrichment analysis of volume specific cytokines showing a >2 fold change was conducted at 10 and 50 weeks. At 10 weeks after irradiation, lung fibrosis was highly enriched following lung irradiation and co-irradiation in agreement with the histological trends. Despite causing high levels of lung fibrosis, this pathway was not enriched for heart-only irradiation at 10 weeks but was enriched at 50 weeks. The impact of heart base irradiation on pericardial and myocardial fibrosis was also reflected by the enrichment of osteopontin (OPN) signalling via the upregulation of MMP-9 as this is associated with cardiac remodelling and fibrosis (42, 43). In contrast, OPN expression was downregulated at 10 weeks after co-irradiation, potentially supporting the observed trend in cardiac functional parameters. Also, enrichment of TGF-β signalling at 50 weeks following heart and lung co-irradiation may be due to the upregulation of MMP-9 which activates TGF-β (44). This also correlates with downregulation of IFN-γ which is suppressed by TGF-β (45). These results correlate with collagen deposition in the right lung following heart and lung combined irradiation. These data highlight the complexity of radiation-induced changes in cytokine signalling for different thoracic volumes and have value in identifying the underlying biological processes involved.

Previously, lung irradiation was found to induce PAH with a dose and volume dependency, with severe pulmonary hypertension associated with a large lung volume (75%) irradiated with a high dose of 17 Gy (14). Our study failed to definitively demonstrate that radiation effects on the lung contribute to cardiotoxicity and is subject to several limitations. CT image guidance was used to irradiate a small lung volume with a dose of 10 Gy. In contrast, most previous studies irradiated lung volumes >25% with doses of 12 and 20 Gy (46) and so the lack of an observed phenotype may be due to the irradiation of a small volume at a subthreshold dose. Also, changes in lung function were not assessed using techniques such as whole-body plethysmography (WBP) and would be informative in understanding the complex interplay following heart and lung irradiations (47). Finally, additional validation of the volume specific cytokine changes is required under different irradiation conditions to demonstrate potential value as biomarkers of response. This study was conducted only in female mice and is subject to sex bias.

Overall, this study presents mild effects after heart and lung co-irradiation. While these results offer limited insight into these effects at lower doses and volumes than previously reported, they map out a potential dose-volume effect for doses similar to clinical dose baths delivered to normal tissues for a thoracic tumour. Despite these limitations, our study raises questions concerning the inter-relationships between lung and heart doses that merits further investigation. Understanding this potential interplay is important in the context of optimising cardiac dose distributions that may increase lung doses and minimising the impact of lung dose on cardiac function.

## Data Availability

Data is available upon request from corresponding author.

## Supplementary material

Supplementary Figure 1 The impact of thoracic irradiation configurations on cardiac systolic and diastolic parameters at 10 weeks after irradiation. **(a)** EF, **(b)** FS, **(c)** E/A Ratio, and **(d)** MPI were evaluated in mice after heart base, heart and lung, and lung only irradiations. Data presented are an average of 6 mice per treatment group ± SEM against age-matched control values. Significance values were classified as **p* < 0.05, ***p* < 0.01 and ****p* < 0.001.

Supplementary Figure 2 Representative images of lung tissue after irradiation showing H&E staining (panel A) and Masson’s Trichrome staining (Panel B).
