# Supplementary figures and images for: Preclinical characterisation of changes in cardiac function and circulating biomarkers following differential irradiation of thoracic volumes
Source: Front Oncol. 2025 Jun 27;15:1623753. doi: 10.3389/fonc.2025.1623753 (PMC12245881; doi:10.3389/fonc.2025.1623753)

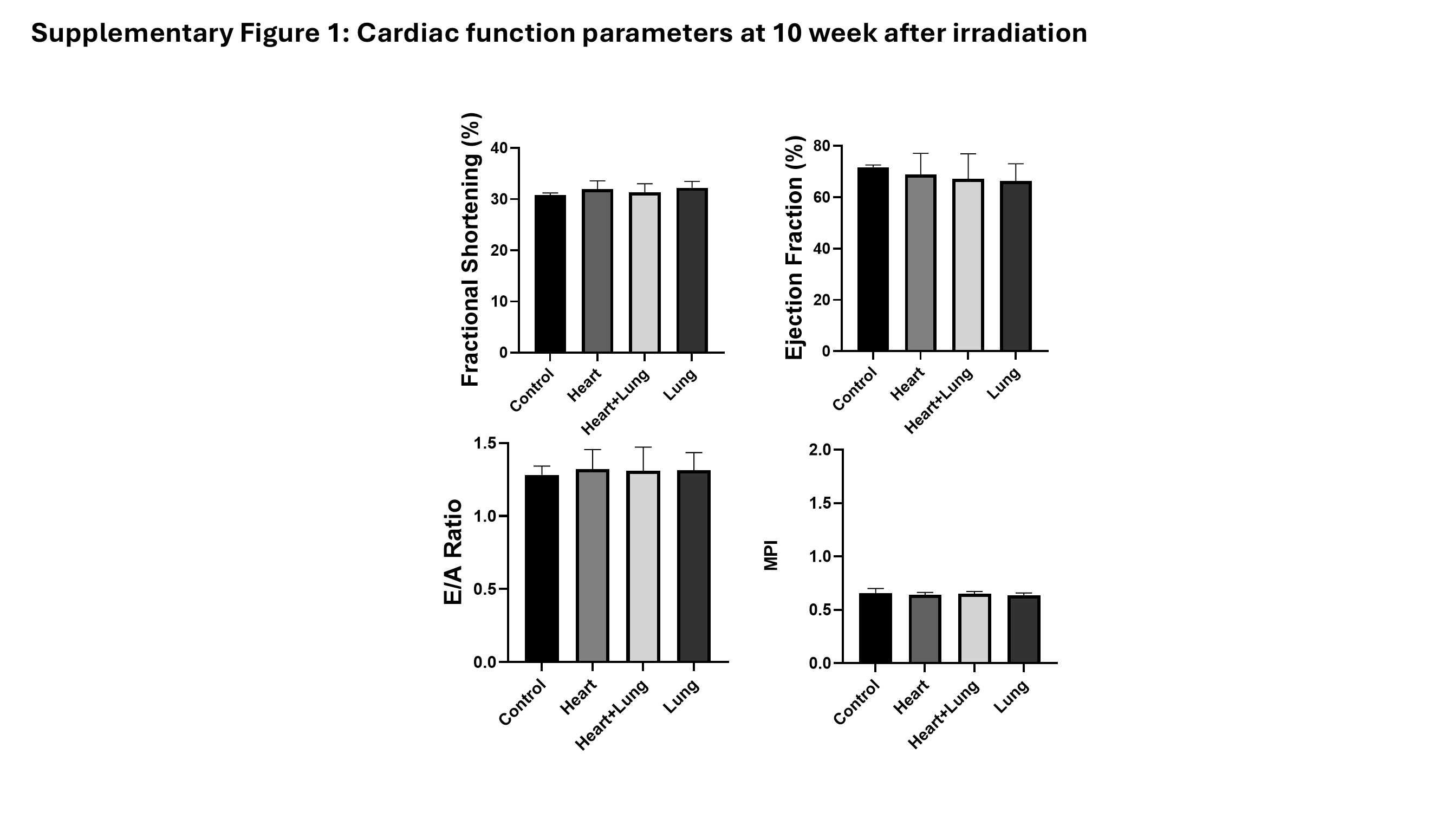

Supplement: Supplementary Figure 1 — The impact of thoracic irradiation configurations on cardiac systolic and diastolic parameters at 10 weeks after irradiation. (a) EF, (b) FS, (c) E/A Ratio, and (d) MPI were evaluated in mice after heart base, heart and lung, and lung only irradiations. Data presented are an average of 6 mice per treatment group ± SEM against age-matched control values. Significance values were classified as *p < 0.05, **p < 0.01 and ***p < 0.001. [file Image1.tif]

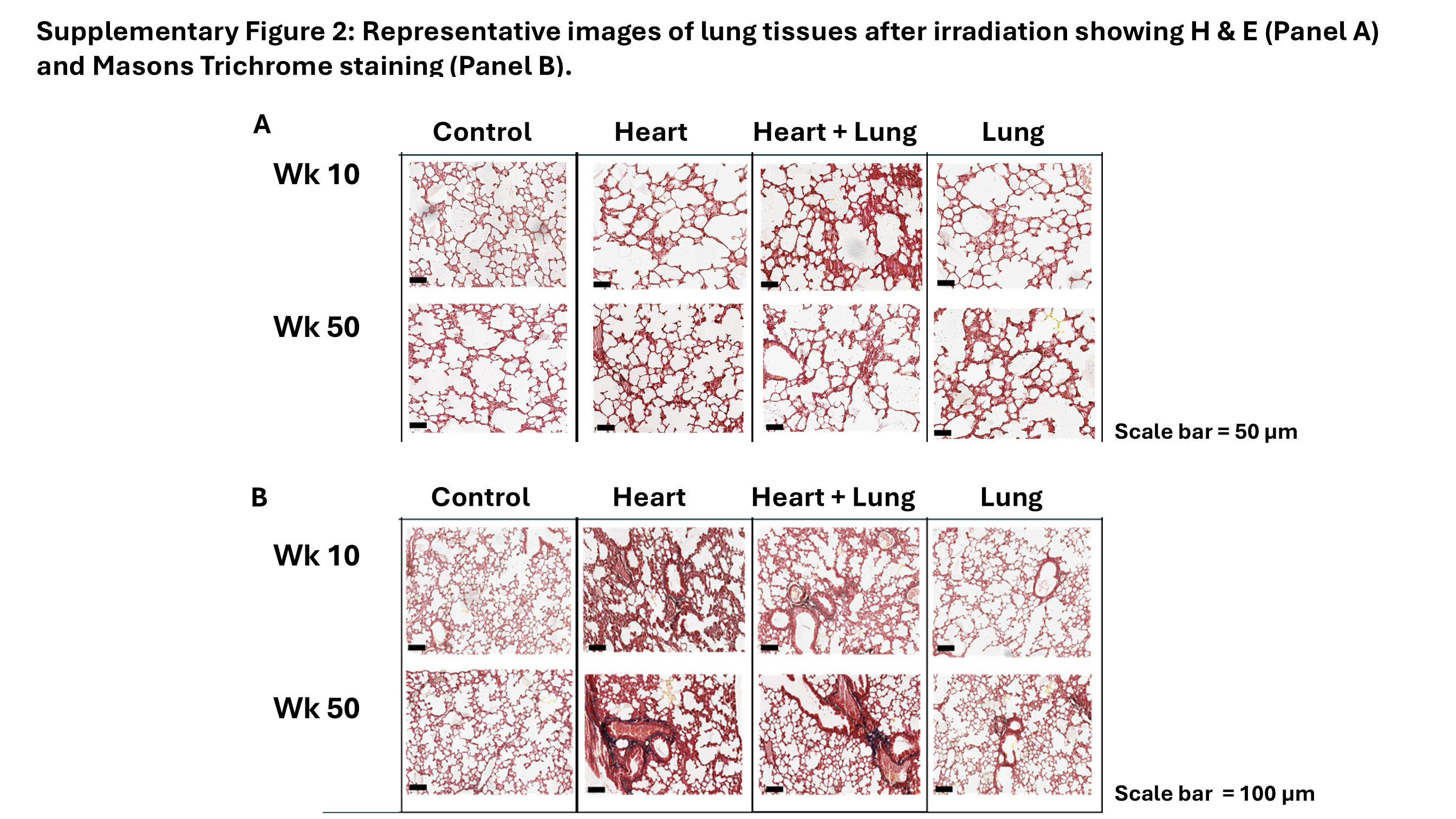

Supplement: Supplementary Figure 2 — Representative images of lung tissue after irradiation showing H&E staining (panel A) and Masson’s Trichrome staining (Panel B). [file Image2.tif]
